# How common is a common error term? The rules that govern associative learning in sensory preconditioning and second-order conditioning

**DOI:** 10.3389/fnbeh.2022.954646

**Published:** 2022-10-14

**Authors:** Travis P. Todd, Nathan M. Holmes

**Affiliations:** ^1^Department of Psychological Science, University of Vermont, Burlington, VT, United States; ^2^School of Psychology, University of New South Wales, Sydney, NSW, Australia

**Keywords:** higher-order conditioning, sensory preconditioning, second-order conditioning, prediction error, Pavlovian conditioning

## Abstract

In standard (first-order) Pavlovian conditioning protocols, pairings of an initially neutral conditioned stimulus (CS) and a biologically significant unconditioned stimulus (US) result in the formation of a CS-US association. The strength of this association is theoretically regulated by prediction error: specifically, the difference between the total level of conditioning supported by the US and the degree to which it is predicted by all stimuli present (i.e., a common error term). In higher-order conditioning protocols (e.g., sensory preconditioning and second-order conditioning), a Pavlovian CS is used to condition responses to other stimuli with which it is paired. At present, it is unknown whether error-correction processes regulate associative learning in higher-order conditioning and, if so, whether these processes are the same as those that regulate formation of a CS-US association in first-order conditioning. Here we review studies that have provided findings relevant to this question: specifically, studies that have examined blocking and/or inhibitory learning in sensory preconditioning and second-order conditioning. These studies show that: (1) animals can form inhibitory associations between relatively neutral sensory stimuli; (2) the learning that occurs in sensory preconditioning and second-order conditioning can be blocked; and, finally, (3) a first-order CS can block conditioning to a second-order CS, and vice versa. The findings are taken to imply that a common error term regulates associative learning in higher-order conditioning, just as it regulates associative learning in first-order conditioning. They are discussed with respect to the nature of the error signal that underlies conditioning and future work that is needed to advance our understanding of the rules that govern different types of learning.

## Introduction

Studies of Pavlovian conditioning have often focused on identifying what is encoded during learning, as well as the circumstances or mechanisms that promote learning (Dickinson, 1980; Rescorla, 1988). In Pavlovian first-order conditioning, it is now generally accepted that organisms encode an association between the conditioned stimulus (CS) and the unconditioned stimulus (US). Such an association can potentially include many features of the US, including its sensory, motivational, temporal, and hedonic properties (Delamater, 2012). One of the greatest insights in Experimental Psychology over the past 50 years is that first-order conditioning is regulated by prediction error, or the discrepancy between what occurs and what is expected; and that all stimuli present at the time of the CS-US pairings contribute to this error calculation. This was formalized in the Rescorla-Wagner model (Rescorla and Wagner, 1972), which states:


ΔV=α×β×(λ−∑V)

According to this formula, the change in the strength of the CS-US association on a given trial (ΔV) is based on the difference between the amount of conditioning supported by the US (λ) and the summed associative strength of all other cues present (ΣV), multiplied by learning rate parameters for the CS (α) and US (β).

**FIGURE 1:**
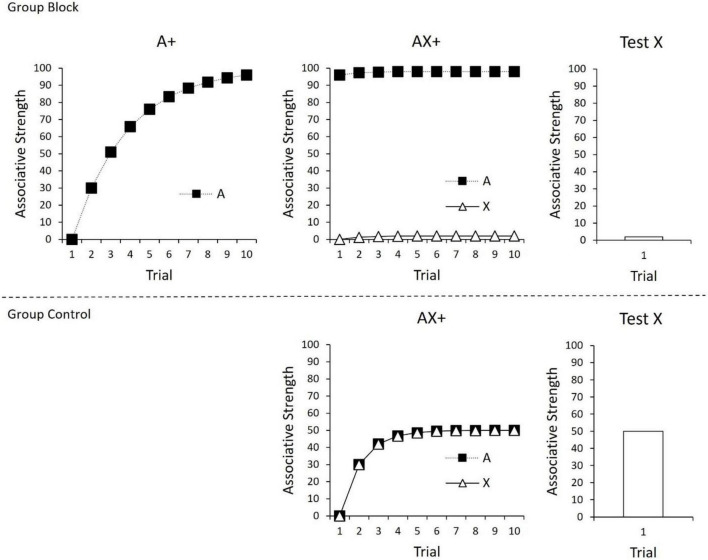
**(Top)** Conditioning of A in Phase 1 **(left panel)**, followed by compound conditioning of AX in Phase 2 (middle panel), and finally a test of X **(right panel)**. As a consequence of prior conditioning to A, X gains little associative strength. **(Bottom)** Compound conditioning of AX+, followed by a test of X. In this case, there was no prior conditioning of A, leading X to gain considerable associative strength. For all panels, changes in associative strength were calculated using the Rescorla-Wagner model (1972), ΔV = α × β × (λ–ΣV), with α × β = 0.3 and λ = 100.

As all cues present on a trial contribute to the prediction error calculation (c.f., Witnauer et al., 2014), the Rescorla-Wagner model is often described as using a “common error term” to calculate associative change. The use of a common error term set the Rescorla-Wagner model apart from its predecessors and enabled it to explain a range of conditioning phenomena at the time of its inception (Miller et al., 1995), including Kamin’s (1969) seminal finding of “blocking.” In the case of blocking, prior conditioning of one CS (A) will interfere with or block conditioning to a novel cue (X) if they are subsequently conditioned in compound. This is depicted in Figure 1; the strength of the CS-US association (“associative strength”) for A is depicted in the top left panel over the course of 10 trials. Initially, the CS has no associative strength, and therefore the difference between V and the level of conditioning supported by the US (i.e., λ−ΣV) is large. However, with continued trials, the associative strength (V) of A continues to increase and approach the maximum level supported by the US (λ), resulting in decreases in prediction error. In the next phase of the experiment (top middle panel), A is presented in compound with a novel cue (X) and reinforced. According to the Rescorla-Wagner model, there should be little prediction error during this phase of the experiment. This occurs because prediction error is based on the *summed* associative strength of all cues present on a trial. Given that A already has a high associative strength (V), when AX is presented, the summed associative strength of A and X (ΣV) already approaches the maximum level of conditioning supported by the US (λ), and therefore the resulting difference between these two values is small (λ−ΣV). As depicted in the top right panel, a final test of X alone reveals that it gained little associative strength over the course of compound training. The associative strength of X in the top panel can be compared to a control group (bottom panel) in which compound training of AX is not preceded by initial training of A. In this case, conditioning of AX endows X with a moderate amount of associative strength.

The Rescorla-Wagner model’s common error term predicts that if the outcome of compound trials differs from what is expected based on the pre-trained stimulus A in Phase 1, the resultant prediction error will ensure that X acquires some conditioning strength. For example, if the magnitude of the US increases for AX conditioning trials during Phase 2, the total level of conditioning supported by the US (λ) will therefore exceed the summed associative strength of A and X, and λ−ΣV will be positive. Thus, the novel stimulus X will acquire positive associative strength. In contrast, if the magnitude of the US were to decrease, or if the US were to be completely omitted, λ−ΣV will be negative, and the novel stimulus will therefore acquire negative or inhibitory associative strength.

Much of what is known regarding the role of prediction error in Pavlovian conditioning is from studies of first-order Pavlovian conditioning, in which the CS is directly paired with the US. However, CSs can also gain the ability to elicit conditioned responses (CRs) through higher-order conditioning, as in sensory preconditioning and second-order conditioning. In these cases, CSs acquire the ability to elicit CRs despite never being directly paired with the US. Studies of higher-order conditioning have often focused on the type of associations that are acquired in sensory-preconditioning and second-order conditioning. Less is known, however, about the role of prediction error in higher-order conditioning. Considering that, in contrast to first-order conditioning, higher-order conditioning does not involve direct CS-US pairings, it is important to consider if the same prediction error mechanisms that govern first-order conditioning also apply to higher-order conditioning. Thus, the purpose of the present paper is to review studies that have examined the role of prediction error in higher-order conditioning. In doing so, we will assess if first- and higher-order conditioning are established via similar or distinct prediction error mechanisms.

## Sensory preconditioning and second-order conditioning

Sensory preconditioning and second-order conditioning are both forms of higher-order conditioning. In each case, conditioned responding develops to a stimulus that was never directly paired with reinforcement. Both forms of higher-order conditioning are robust in the sense they have been observed across a variety of conditioning protocols and species (e.g., Brogden, 1947; Rizley and Rescorla, 1972; Rescorla, 1984; Nicholson and Freeman, 2000; Dwyer et al., 2012; for a review, see Holmes et al., 2022).

In the case of sensory preconditioning, two cues are first presented in compound (S2 and S1), after which one of the cues (S1) is paired with reinforcement. The initial pairings of S2 and S1 are thought to establish an association between the two (Rizley and Rescorla, 1972). Given this, there are at least two ways by which S2 can then gain the ability to elicit a CR at test (for a review see Holmes et al., 2021). One possibility is that S2 elicits a CR at the time of test through an associative chain. In this case, presentation of S2 activates the representation of S1, which activates the representation of the US (Rescorla and Cunningham, 1978; Rescorla and Freberg, 1978; Sharpe et al., 2017a). A second possibility is that during S1-US pairings, S1 retrieves the memory of S2, allowing S2 to become associated with the US through mediated conditioning (Rescorla and Cunningham, 1978; Rescorla and Freberg, 1978; Wong et al., 2019). In a recent review, Holmes et al. (2021) noted that there is evidence in favor of both types of integration, and the type of integration that occurs may depend on a variety of parameters and training/test conditions.

In studies of second-order conditioning, S1 is first paired with reinforcement and is then subsequently paired with S2 (typically without the original reinforcer). There are at least three possible associative structures that can, in principle, support responding to S2 during second-order conditioning (Rizley and Rescorla, 1972; Seitz et al., 2021). One possibility is that S2 and S1 become directly associated with each other: hence, responding to S2 at test is governed by an associative chain, S2 → S1 → US. A second possibility is that during pairings of S2 and S1, the latter activates the representation of the US (due to previous conditioning of S1) which is then associated with S2. In this case, S2 is associated with the US through mediated conditioning. Finally, a third possibility is that during pairings of S2 and S1, S2 becomes associated with either the central motivational state or responses evoked by S1 (Rescorla, 1973, 1974, 1982). In this case, responding to S2 is conceptualized as an S-R association.

### Prediction error and sensory preconditioning

#### Excitatory associations involving neutral stimuli

In contrast to first-order conditioning, which often involves a biologically significant US (e.g., footshock, food pellets), the stimuli used in phase 1 of sensory preconditioning typically have little or no biological significance. For this reason, phase 1 of sensory preconditioning constitutes an ideal testing ground for assessing whether a common error term regulates the acquisition of associations between relatively neutral sensory stimuli.

Sharpe et al. (2017b) utilized a within-subject version of sensory preconditioning to assess whether acquisition of a preconditioned association between a novel stimulus (e.g., C) and X can be blocked by a pretrained signal for X, in the same way that the directly conditioned association between X and the US can be blocked by a pretrained signal for the US (see Table 1). In phase 1, hungry rats were first exposed to trials on which A appeared and was followed by presentation of X. Then, while A-X pairings continued, rats were additionally exposed to trials on which the presentation of X was preceded by a simultaneous compound of two stimuli (one auditory and one visual), including: (1) the pre-trained A and a novel stimulus, C [i.e., AC-X]; (2) the pre-trained A and a novel stimulus, D [i.e., AD-X]; and, finally, (3) two novel stimuli, E and F [EF-X]^1^. In phase 2, rats were exposed to pairings of X and a food pellet US. Finally, in phase 3, rats were tested with presentations of the target stimuli C, D and F, and food cup entries during cue presentations were monitored as the conditioned response. Sharpe et al. (2017b) reasoned that, if acquisition of the C-X and D-X associations in phase 1 was blocked by the pre-trained A, then during testing in phase 3, C and D should be less effective in activating the representation of X and, thereby, the US relative to test presentations of F, for which the association with X was not blocked: hence, C and D should elicit less responding than F. This was exactly the result obtained and was taken to imply that the association between two affectively neutral stimuli is subject to blocking in the same way as the association between a CS and US; and, hence, that the association between such sensory stimuli is regulated by a common error term in the same way as the association between a CS and US.

**TABLE 1:** Blocking during sensory preconditioning.

Sharpe et al. (2017b)			

Preconditioning		Conditioning	Probe test
A → X	EF → X AD → X AC → X A → X	X → US	F D C

Blocking during the preconditioning phase of sensory preconditioning (Sharpe et al., 2017b). The design is fully within-subject. Thus, each subject received all trial types listed for each phase. During the probe test, rats responded more to F than D and C. A and E are visual cues, C, F, D, and X are auditory cues.

#### Inhibitory associations involving affectively neutral stimuli

Espinet et al. (2004) also provided evidence that associations established during sensory preconditioning are regulated by a common error term. However, rather than examining blocking of sensory preconditioning, these authors examined whether rats form inhibitory associations between affectively neutral stimuli: i.e., whether a neutral visual stimulus, X, could enter into an inhibitory association with a neutral auditory stimulus, B. In each of two experiments (see Table 2), all rats were exposed to trials on which an auditory stimulus, A (a tone), was presented in simultaneous compound with a visual stimulus, X (a key light by the magazine). For rats in one group (Paired), the AX- trials were intermixed with exposures to forward serial pairings of the stimuli A and B (A→B; B = a white noise). For rats in another group (Unpaired), the AX- trials were intermixed with exposures to explicitly unpaired presentations of A and B (A/B). If associations learned during sensory preconditioning are regulated by a common error term in the same way as associations learned during first-order conditioning, then an inhibitory association would be expected to develop between X and B in Group Paired but not in Group Unpaired. Specifically, in the former group, pairings of A and B would produce a positive association between these two stimuli. Then, on AX- trials, the summed associative strength (ΣV) for the prediction of B would also be positive. However, because B is not presented on AX- trials, λ_*B*_ would be zero, and the resulting prediction error would result in X gaining negative associative strength with respect to B. This, of course, would not occur for rats in Group Unpaired as, on AX- trials, the summed associative strength for the prediction of B would be zero (as A and B were never paired), meaning that X would not enter into any sort of relation with B.

**TABLE 2:** Inhibitory sensory preconditioning reported by Espinet et al. (2004).

Experiment 1				

Group	Pre-exposure	Conditioning	SPC Test	Retardation
Paired	A → B, AX	B → food	A, AX	X → food
Unpaired	A, B, AX	B → food	A, AX	X → food

**Experiment 2**				

**Group**	**Pre-exposure**	**Conditioning of B**	**Conditioning of C**	**Summation**

Paired	A → B, AX	B → food	C → food	C, CX
Unpaired	A, B, AX	B → food	C → food	C, CX

Experiment 1: Conditioning to X was slower during the retardation test for Group Paired compared to Group Unpaired. Experiment 2: Summation to CX was stronger for Group Paired relative to Unpaired.

To determine whether rats in Group Paired learned an inhibitory X-B association, subsequent to the training described above, Espinet et al. (2004) conditioned all rats to approach the food cup/magazine during pairings of B with a food pellet US; and then exposed them to a series of tests. In Experiment 1, rats were tested with presentations of A alone and trials on which X was itself paired with the food pellet US. In Experiment 2, rats were tested with presentations of a separately established excitor, C (a house light located on the wall directly opposite the magazine) and, critically, X in compound with this excitor, CX-. Espinet et al. (2004) expected that rats in Group Paired would exhibit sensory preconditioned responding during the test presentations of A alone: i.e., that they would integrate the A→B and B→food associations formed in training to respond when tested with A, and that this level of responding would be greater than that exhibited by rats in Group Unpaired, as the latter had received explicitly unpaired exposures to A and B in the initial training. This was not quite borne out in their data: both groups responded equally when tested with A, which was attributed to generalization between the auditory stimuli (i.e., from B to A) in Group Unpaired. Nonetheless, the question of interest concerned the relative level of responding in these two groups during the final series of X-food pairings (Experiment 1), which was effectively a retardation test for the putative inhibitory relation between X and B; and during the test presentations of C- and CX- (Experiment 2), which was effectively a summation test for the same inhibitory relation.

Espinet et al. (2004) reasoned that, if rats in Group Paired had encoded an inhibitory association between X and B in the initial phase of training, then at the time of testing, X would inhibit the representation of B as well as that of its associates, including the food pellet US. Accordingly, these authors predicted that, relative to rats in Group Unpaired, rats in Group Paired would be slower to acquire conditioned responding to X when it was directly paired with the food pellet US (retardation test) and show a greater reduction of responding when X was combined with the separately established excitor, C (summation test). Both predictions were confirmed: rats in Group Paired responded less than rats in Group Unpaired in the retardation test and exhibited a greater difference in responding to C and CX in the summation test. Hence, Espinet et al concluded that rats in Group Paired had acquired an inhibitory association between X and B, thereby imbuing X with the capacity to inhibit the representation of the food pellet US. The important corollary of this result is that associative learning in sensory preconditioning is regulated by a common error term. That is, the discrepancy between the expectancy of B and its absence on AX- trials was shared between A and X; thus allowing X to acquire negative or inhibitory strength with respect to B and, thereby, pass the retardation and summation tests for this inhibition.

### Prediction error and second-order conditioning

As we have noted, the observation of blocking in first-order conditioning is consistent with the idea that learning is regulated by a common error term (Rescorla and Wagner, 1972; cf. Mackintosh, 1975). If second-order conditioning is also driven by a common error term, then second-order conditioning of one stimulus would be expected to block second-order conditioning to a new stimulus. A small set of studies have confirmed this prediction. Leyland and Mackintosh (1978) observed blocking of second-order conditioning in a pigeon autoshaping procedure. In phase 1 of their experiment, pigeons received pairings of a red keylight with food. In phase 2, second-order conditioning was established to one of two non-localized stimuli (either an auditory click or a diffuse houselight, counterbalanced) via pairings with the red keylight. In phase 3, a white keylight was then compounded with the non-localized cue and followed immediately by the red keylight. For one group of birds (Group Block, “B”) the compound in phase 3 consisted of the non-localized cue that underwent second-order conditioning in phase 2. For a second group (Group Control “C”), the compound consisted of the white keylight and the non-localized cue that had not been conditioned. Leyland and Mackintosh (1978) found that responding during second-order conditioning developed more rapidly in Group C than Group B, indicating blocking of second-order conditioning to the white keylight by prior second-order conditioning to the non-localized stimulus.

**FIGURE 2:**
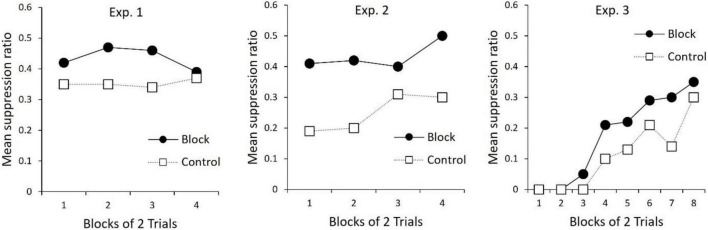
Re-drawn results from Bombace (1982). All panels depict suppression to the Light during the Test phase (see Table 3). For Experiment 1, Bombace (1982) reports that suppression to the Light in Block was significantly less than Control on trial blocks 1–4. For Experiment 2, Bombace (1982) reports suppression to L was less for Block than Control on trial blocks 1–4. For Experiment 3, suppression in Block was less than Control on trial block 5, and over trial blocks 7–8.

An unpublished doctoral dissertation by Bombace (1982; see Table 3) reported a series of experiments examining blocking of second-order conditioning with aversive conditioning procedures. In Experiment 1, all rats received first-order conditioning by pairing a tone with mild foot-shock. For one group of rats, second-order conditioning was then established to a noise by pairing it serially with the tone. A control group received unpaired presentations of the noise and tone. In the next phase, a light cue was followed serially by the first-order tone to establish second-order conditioning of the light. However, for all rats, the noise was also presented in compound with the light. Thus, for one group of rats, second-order conditioning to the light occurred in the presence of a previously trained second-order stimulus. For the second group of rats, the added cue (noise) did not have this prior treatment. The final test of the light revealed less second-order conditioning for the group in which the noise had been pre-trained as a second-order stimulus (results re-drawn in Figure 2). Thus, replicating the findings of Leyland and Mackintosh (1978), second-order conditioning of one stimulus blocked second-order conditioning to a new stimulus (see also Zimmer-Hart, 1974). Together these findings indicate that, like first-order conditioning, second-order conditioning is regulated by prediction error that includes a common error term.

**TABLE 3:** Blocking experiments reported by Bombace (1982).

Group	Pre-test 1	FOC	SOC	Pre-test 2	Blocking	Test
**Experiment 1: Blocking of 2nd order by 2nd order**						
Block	L-	T + , N-	T + , N→T	L-	T + , NL→ T	L-
Control	L-	T + , N-	T + , N/T	L-	T + , NL→ T	L-
**Experiment 2: Blocking of 2nd order by 1st order**						
Block	L-	T + , N +		L-	T + , NL→ T	L-
Control	L-	T + , N-		L-	T + , NL→ T	L-
**Experiment 3: Blocking of 1st order by 2nd order**						
Block	L-	T + , N-	T + , N→ T	L-	T + , NL +	L-
Control	L-	T + , N-	T + , N/T	L-	T + , NL +	L-

Prior to Pre-Test 1 all rats received magazine training followed by lever-press training. “FOC” = first-order conditioning, “SOC” = second-order conditioning. In Experiments 1 and 2, “-” indicates no reinforcement, “ + ” is a 0.5-s, 0.5-mA footshock. In Experiment 3, “ + ” was a 0.5-s, 1-mA footshock during FOC and 0.5-s, 0.5-mA during blocking. This change in shock value was an attempt to equate the value of the reinforcer in the Blocking phase with the previously acquired value of the second-order reinforcer (Bombace, pg. 50). For Experiment 2, Bombace (1982) also included a group to test if first-order inhibition would produce second-order super conditioning. There was no evidence of super-conditioning, however, Bombace speculated that “perhaps the blocked cue (N) was not inhibitory” (pg. 47). For brevity, this group is not depicted in the table or figure. In Experiment 3, Bombace (1982) included two groups that replicated the finding that second-order conditioning blocks subsequent second-order conditioning. These groups are not depicted in the table or figure.

In further examination of the processes that regulate first- and second-order conditioning, Bombace (1982) assessed blocking across orders of conditioning. The design of these experiments is presented in Table 3. In Experiment 2, two group of rats received first-order conditioning with a tone. Rats in one group (Block) also received first-order conditioning with a noise, but for rats in Group Control the noise was presented and non-reinforced. In the next phase, for all rats the noise was compounded with a light and followed serially by the tone. This was done to (potentially) establish second-order conditioning of the light by the tone. During a final test of the light, there was more suppression for Group Control than for Group Block (middle panel Figure 2). Thus, first order conditioning of the noise for Group Block, impaired second-order conditioning to the light.

Experiment 3 tested the opposing interaction between orders of conditioning; the ability of prior second-order conditioning to block first-order conditioning. In this experiment (see Table 3), one group of rats received prior second-order conditioning of a noise (Group Block), whereas the control group received a treatment intended to keep the noise relatively neutral (Control). All groups then received first-order conditioning of a noise-light compound. During the final test of the light, there was more suppression for Group Control relative to Group Block. Thus, second-order conditioning was found to block first-order conditioning. Together with the findings described above, these results show that, not only are the two types of conditioning regulated by a common error term, but additionally, the error signal that regulates the two types of conditioning is coded in common terms (see also Jones et al., 2012, for evidence that a stimulus that elicits sensory preconditioned responding can block first-order conditioning to a new stimulus). This is considered further in the next section.

## Common error in higher-order conditioning: Additional implications

The studies reviewed here suggest that, like the learning that occurs in first-order conditioning, the learning that occurs during higher-order conditioning is regulated by a common error term. They also shed light on specific features of such learning. For example, the first- and second-order conditioning procedures used in the experiments reported by Bombace (1982) establish different associations. This follows from studies by Rescorla who showed that, following CS-US pairings in first-order conditioning, habituation of the US reduced fear to the CS, indicating that this fear is indeed regulated by a CS-US association (Rescorla, 1973). By contrast, following serial pairings of S2 and an already-conditioned S1 in second-order conditioning, habituation of the US had no impact on fear to the S2, suggesting that this fear is likely supported by some form of S-R association (i.e., during S2-S1 pairings, S2 associated with the response evoked by S1; Rescorla, 1973). Thus, in showing that blocking can occur across orders of conditioning, the experiments by Bombace suggest that a CS-US association can block formation of an S-R association and vice versa, raising the question of how a common error term operates to regulate the interaction between first- and second-order CSs; or, more specifically, what is shared between first- and second-order conditioning on which a common error term operates.

One possibility is that a common error term operates at the level of the CR. In this case, error occurs when there is discrepancy between the CR elicited, and the maximum level of the CR that can be supported by the respective reinforcer. Such response error, with respect to first-order conditioning, was initially discussed by Rescorla and Wagner (1972) and has more recently been emphasized in instrumental learning (Bouton et al., 2021). Applied to the experiments reported by Bombace (1982), blocking across orders of conditioning occurs because the strength of the CR elicited during compound training is already maximally elicited by the pretrained CS. Thus, in this case there is no discrepancy between the CR elicited on compound trials and the CR that is already supported by the pretrained first- or second-order CS.

A second possibility is that common error operates at the level of affect. As we have noted, Pavlovian conditioning establishes associations between the CS and several aspects of the US, including its sensory and affective qualities (Wagner and Brandon, 1989). With respect to cross-order blocking, although the sensory features of the reinforcer presumably change across each stage of training, the affective state elicited by each stimulus is likely shared. As noted by Bombace (1982), in this case the prediction error would be determined by the difference between the current level of affect elicited by all cues present, and the total level of affect supported by the reinforcer (see also Ganesan and Pearce, 1988). The notion that prediction error operates at the level of affect is consistent with evidence from first-order conditioning. For example, Ganesan and Pearce (1988) observed blocking when the reinforcer was changed from water in the first phase to food in the second phase. In this case, although the specific sensory features of the reinforcer changed from phase 1 to phase 2, the common affective (in this case appetitive) properties did not (see also Bakal et al., 1974; Betts et al., 1996; see Rescorla, 1999 for evidence of learning about different outcome during a blocking procedure). Indeed, Ganesan and Pearce (1988) favored blocking occurring at the level of affect as opposed to the response, because blocking was observed even when different responses were required (Ganesan and Pearce, 1988, Experiment 4).

The idea that a common error term operates at the level of the CR or affect can account for most of the findings relating to second-order conditioning. It is not, however, readily suited to explain the role of prediction error during sensory preconditioning, in which there is no overt CR and the stimuli are relatively neutral. Instead, it is likely that prediction error occurs with respect to the sensory features of the cues that are being associated. Thus, while the overall pattern of results suggests that higher-order conditioning is regulated by a common error term, the error signal might reflect sensory processing during sensory preconditioning, and response or affect processing in second-order conditioning. Although this requires further testing, the idea of multiple learning processes operating in higher-order conditioning is consistent with some approaches to first-order conditioning that postulate learning about distinct components of the US (e.g., Wagner and Brandon, 1989; see Delamater, 2012).

## Limitations

Higher-order conditioning was first-documented by Pavlov (1927) and the Rescorla and Wagner (1972) model was published 50 years ago. Nonetheless, few studies have provided evidence relevant to the question of whether higher-order conditioning is regulated by a common error term. The few studies that have provided such evidence are those by Sharpe et al. (2017b), Espinet et al. (2004), Leyland and Mackintosh (1978), and Bombace (1982). These studies are elegant in their design and we have taken their results to suggest that associative learning in higher-order conditioning is, indeed, regulated by a common error term. However, it is important to note the potential limitations of these studies.

In the study by Sharpe et al. (2017b; blocking of sensory preconditioning), the final series of tests revealed that rats responded less to C that had been initially presented in compound with the familiar stimulus A (A→X trials preceded AC→X trials) than F that had been initially presented in compound with the novel stimulus E (on EF→X trials); and this was taken to mean that acquisition of the C→X association was blocked by A relative to acquisition of the F→X association that was not blocked by E. However, as A and E differed in their familiarity during their compounding with C and F, respectively, the pattern of test responding to C and F can be explained without reference to blocking of the C-X association by A. Instead, one need only assume that some of the responding to C and F reflects generalization from X; and that the attention commanded by F, which had been presented in compound with the novel E, was greater than the attention commanded by C, which had been presented in compound with the familiar A (for a similar argument with respect to latent inhibition, see Hall and Rodriguez, 2010, 2011). Hence, F was better able to elicit/control responding than C/D at test.

In the study by Espinet et al. (2004; inhibitory sensory preconditioning), after the initial stage of training in which the putative inhibitory X-B association was established, rats were conditioned to B and then subjected to analogs of the standard retardation and summation tests for inhibitory learning to X: the supposition being that an inhibitory X-B association would enable X to pass such tests. The results were generally consistent with this supposition; but one may ask why it was supposed in the first place. That is, there is no obvious reason why an inhibitory X-B association would interfere with acquisition of an X-food association; unless it is additionally supposed that X inhibits B *as well as B’s associates* – which, to the best of our knowledge, has not been demonstrated.

These issues in relation to the Sharpe et al. (2017b) and Espinet et al. (2004) studies are not intended to challenge the conclusions of those studies or the claim that associative learning in higher-order conditioning is regulated by a common error term. Rather, we cite these issues as a means of highlighting the need for further work to more firmly establish inferences drawn from this small number of studies. To be perfectly clear, we take the *collection* of results provided by Sharpe et al. (2017b) and Espinet et al. (2004) as sufficient evidence that acquisition of excitatory and inhibitory associations in sensory preconditioning is regulated by a common error term, especially as it is generally consistent with evidence from other sources. For example, when rats are given intermixed exposures to flavor compounds AX and BX, they form mutually inhibitory associations between the unique features A and B (Dwyer et al., 2001). Hence, associative learning between relatively neutral flavor stimuli appears to be regulated by a common error term; making it likely that the learning of excitatory and inhibitory associations in sensory preconditioning is also regulated by a common error term.

Finally, it is also worth noting that, in the study by Bombace (1982), an established second-order CS blocked *de novo* first-order conditioning to a novel stimulus; and, conversely, an established first-order CS blocked *de novo* second-order conditioning to a novel stimulus. While these results are consistent with the *trans*-reinforcer blocking effect reported by Ganesan and Pearce (1988) in an appetitive conditioning preparation, Bombace did not assess any potential unblocking of the novel stimulus in these cross-order groups (it would have required a different set of controls). Such an assessment would have permitted more specific claims regarding the involvement of error correction processes in higher-order conditioning: e.g., the phenomena of unblocking and “blocking of unblocking” can be used to determine whether error impacts higher-order conditioning directly by regulating how much is learned about stimuli present on the current trial, or indirectly via changes in attention to stimuli on subsequent trials (see Fam et al., 2017 for a discussion of these phenomena in relation to first-order conditioning). Instead, such claims await further research.

## Conclusion: Common error as common ground for first- and higher-order conditioning

The findings reviewed here suggest that a common error correction process appears to be common to all types of associative learning in first- and higher-order conditioning protocols. That is, a common error term regulates learning about the relations between neutral stimuli in sensory preconditioning and operates at the level of the response and/or affect to regulate the learning that occurs in second-order conditioning. This conclusion will be strengthened by additional studies that continue to examine the role of error-correction in higher-order conditioning across a range of parameters and procedures. In particular, future studies should address: (1) how a common error term regulates associative learning with neutral stimuli (2) the generality of the Bombace findings to other aversive protocols (e.g., conditioned freezing) and appetitive conditioning; and (3) the possibility that a common error term also regulates the extinction of higher-order conditioning in the same way that it has been shown to regulate extinction of first-order conditioning (e.g., Leung and Westbrook, 2008; Leung et al., 2012; Holmes and Westbrook, 2013). Such studies are needed to determine the ubiquity of a common error term in regulating associative learning across all types of learning.

## Author contributions

Both authors contributed to the article by conceiving the idea and writing the manuscript, and approved the submitted version.

## Footnotes

1 In the design by Sharpe et al. (2017b), the AC → X and AD → X trials are functionally equivalent. The reason why both sets of trials were included is that Sharpe et al. (2017b) went on to examine if activation of ventral tegmental (VTA) neurons could drive associative formation that would otherwise be blocked. Thus, in one group of rats, VTA neurons were optogenetically activated during AC → X trials, and not AD → X trials. Here we focus on the results of the control group which did not receive VTA activation on any trials.
